# Transcriptional Analysis of the Pre-Erythrocytic Stages of the Rodent Malaria Parasite, *Plasmodium yoelii*

**DOI:** 10.1371/journal.pone.0010267

**Published:** 2010-04-21

**Authors:** Calvin T. Williams, Abdu F. Azad

**Affiliations:** Department of Microbiology and Immunology, University of Maryland School of Medicine, Baltimore, Maryland, United States of America; INSERM U567, Institut Cochin, France

## Abstract

The molecular biology of the clinically silent pre-erythrocytic stages of mammalian *Plasmodium* spp, composed of both the sporozoite and liver stages, has remained largely uncharacterized. Improved understanding of the biological processes required for progression through the pre-erythrocytic stages could lead to the identification of novel drug and vaccine targets. To gain insights into the molecular events that occur during the pre-erythrocytic stages of *Plasmodium*, comparative transcriptional analysis was performed on radiation attenuated sporozoites (RAS), wild type sporozoites (wtSPZ) and liver stage parasites collected either 24 hours (24hrLS) or 48 hours (48hrLS) after mice were infected with *Plasmodium yoelii*. Our results revealed 1100 *Plasmodium* genes that were differentially expressed in one or more constituents of the pre-erythrocytic stages relative to the mixed blood stages. Overall, the transcriptional profile of *P. yoelii* gradually became more similar to the mixed blood stages as pre-erythrocytic stage development progressed into the mature liver stage schizont. The transcriptional profiles of RAS and wtSPZ were found to be nearly identical. Likewise, the transcriptional profile of 24hrLS was very similar to that of the 48hrLS parasites. The largest differences in gene expression were observed when comparing wtSPZ or RAS to either of the liver stage samples. Further characterization of the differentially expressed genes identified in this study could help elucidate the biological mechanisms employed by *Plasmodium* during the pre-erythrocytic stages.

## Introduction

Malaria, caused by hematoprotozoan parasites of the genus *Plasmodium,* remains a global health crisis with high annual morbidity and mortality. The complexity of *Plasmodium's* life cycle hampers efforts to eliminate or significantly reduce its morbidity and mortality, especially within tropical areas of the world. The malaria infection is initiated after *Plasmodium* sporozoites are injected of into the skin during the taking of a blood meal by *Anopheles* mosquitoes. The sporozoites migrate to the liver, invade host hepatocytes, and develop into trophozoites. After a period of growth and several rounds of asexual replication, the parasite transitions into mature liver stage schizonts which contain thousands of infectious merozoites. Ultimately, merozoites are release into the bloodstream and subsequently invade red blood cells. The pre-erythrocytic stages of *Plasmodium*, which consist of the sporozoite and liver stages, represent the first opportunity the mammalian host has to mount a defense against *Plasmodium* infection. Yet in malaria endemic areas, in which frequent exposure to *Plasmodium* sporozoites occur, immune responses directed at these parasite stages are weak, if at all detected [1], [2].

In contrast, robust immune responses directed at the pre-erythrocytic stages of *Plasmodium* was evidenced first in avian malaria [3], [4], then in rodents [4] and finally in humans [5]–[8], using radiation attenuated sporozoites (RAS). The immune response to the pre-erythrocytic stages appears to be elicited by sporozoites in the lymph nodes draining the site of infection[9]. Once activated, cytotoxic T-cells target infected hepatocytes [10], eliminating *Plasmodium* contained within. Further characterization of RAS within the mammalian host revealed that RAS maintained the ability to invade hepatocytes but could not complete the normal intrahepatic development and thus, were incapable of generating infectious merozoites [11]. The molecular basis for the defects responsible for the developmental arrest of RAS within hepatocytes as well as the identity of the antigens involved in the protective immune response directed at the pre-erythrocytic stages remain unknown.

Developing effective pre-erythrocytic malaria vaccines targeting either the sporozoites or the liver stage parasites require a better understanding of how the parasite is able to reside within the mammalian host for days (rodent) or weeks (human) without eliciting strong immune responses. Furthermore, deciphering the molecular events underlying parasite immune evasion during these clinically silent stages of malaria infection can only be achieved by overcoming the technical hurdles inherent to post sporozoite and early liver stage infection. In this work, RNA was collected from RAS and wtSPZ from the salivary gland of mosquitoes infected with the rodent malaria parasite, *P. yoelii*. Also, laser capture microdissection (LCM) was employed to acquire highly pure parasite RNA from the liver stages of *P. yoelii*. Linear amplification of RNA was used to obtain enough material to perform microarray based transcriptional analysis of the pre-erythrocytic stages. The comparative analysis of the transcriptional profiles of the pre-erythrocytic stages identified genes that were differentially expressed, shedding light on the molecular processes involved during the parasite transition from sporozoite to early and late liver stage schizonts.

## Results

### Microarray analysis

To develop a better understanding of the dynamics of the molecular processes occurring during the pre-erythrocytic stages of *Plasmodium* we performed a microarray based analysis of the transcriptional profile of wild type salivary gland sporozoites (wtSPZ), radiation attenuated sporozoites (RAS), and liver stage parasites collected from mice livers 24 hours (24hrLS) and 48 hours (48hrLS) after wtSPZ infection, using the *P. yoelii* (17XNL) rodent malaria model. Sampling was done over a two-year period due to the inherent difficulties of collecting highly pure parasite liver schizonts using LCM. Therefore, a reference design was chosen to allow comparisons to be made between pre-erythrocytic RNA samples hybridized at different times to oligonucleotide arrays which were designed using the genome of *P. yoelii*. All collected wtSPZ, RAS and liver stage samples were compared to the same RNA stock collected from *P. yoelii* mixed blood stages. Using this approach, we were able to reproducibly detect signal from ∼1600 genes in one or more pre-erythrocytic stage constituents.

To identify genes differentially expressed during the pre-erythrocytic stages relative to the mixed blood stages, Statistical Analysis of Microarray [12]–[14] analysis (SAM) was performed. We were able to identify 1133 genes, listed in Table S1, which were significantly differentially expressed when compared to the mixed blood stages, in one or more of the *P. yoelii* pre-erythrocytic samples probed. The number of genes differentially expressed during the pre-erythrocytic life cycle relative to the blood stage parasite were 636, 889, 484 and 271 for wtSPZ, RAS, 24hrLS and 48hrLS respectively and included 117 genes significantly differentially expressed in all pre-erythrocytic samples probed. The majority (115 of 117) of these commonly differentially expressed genes were up regulated throughout the pre-erythrocytic stages relative to the mixed blood stages (listed in Table S1). These 117 genes encoded proteins involved in variety of functions (Figure S1) including cell structure (2), cell invasion (2), protein folding (2), translation (3), transporters (4), transcription (6), and cell development (8). Sixty seven percent of all the commonly differentially expressed pre-erythrocytic genes encoded hypothetical proteins (Table S1). However, no specific pathway encompassing all of the commonly differentially expressed genes could be discerned. (Note: The number in parenthesis following a functional group name represents the number of differentially expressed genes belonging to that functional group)

### Functional Categories

**Figure 1:**
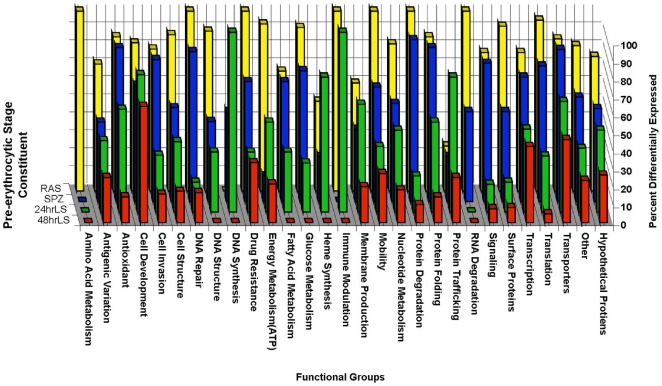
Functional grouping of genes differentially expressed during the pre-erythrocytic stage. All genes identified using significance analysis for microarray (SAM, FDR <1%) to be differentially expressed relative to the mixed blood stages were grouped based on the annotated functions of the proteins they encode. The percent of the total number of genes identified to be differentially expressed at any point during the pre-erythrocytic stage relative to the mixed blood stages that belong to an individual functional group (listed on the x-axis) that were differentially expressed in a particular pre-erythrocytic stage constituent (listed on the z-axis: RAS, yellow bars; wtSPZ, blue bars; 24hrLS, green bars; or 48hrLS, red bars) was plotted on the y axis.

Parasite life cycle progression, after entry into the host skin, journey to the liver and the infection of hepatocytes, undoubtedly require multiple cellular functions to be performed by the parasite for successful completion. These biological processes are likely reflected in the gene expression profile corresponding to each pre-erythrocytic stage. Likewise, variations in the conduction biological processes observed in pre-erythrocytic development compared to the development of plasmodium during the blood stage are likely to be reflected in the differences in the transcriptional profile of the pre-erythrocytic stages compared to the mixed blood stage. To develop a better understanding of the molecular processes that are important for progression through the pre-erythrocytic stages compared to blood stage development, all genes differentially expressed in each pre-erythrocytic stage constituent relative to mixed blood stages were grouped based on the annotated functions of their encoded proteins (Figure 1, detailed in Table S1). The largest functional category for each pre-erythrocytic stage constituent consisted of genes encoding hypothetical proteins (51–60% of all genes differentially expressed by each pre-erythrocytic stage constituent) for which no functional information could be obtained. The second largest group was the “other” functional category. This group is composed of genes encoding proteins with annotated functions or possessing conserved domains implicated in multiple biological processes. For example, although several genes encode proteins with zinc finger motifs (such as PY03958 and PY04485), which are involved in DNA binding, the functional consequences of the binding of DNA by these proteins was not able to be determined solely from the available annotations.

Next, to graphically represent the distribution of the differentially expressed genes within each functional group among the different constituents of the pre-erythrocytic stages the percent of the total number of genes differentially expressed at any point during the pre-erythrocytic stage relative to the mixed blood stages that belong to an individual functional group (listed on the x-axis) that were differentially expressed in a particular pre-erythrocytic stage constituent (listed on the z-axis) was plotted on the y axis in Figure 1. For most functional groups, the number of differentially expressed genes decreased as the pre-erythrocytic stages progressed from sporozoite, wtSPZ and RAS, to 24hrLS followed by 48hrLS. Notable exceptions to this general trend included genes involved in cellular development, which were composed mostly of genes encoding cell cycle regulators or proteins with homeodomains, are differentially expressed throughout the pre-erythrocytic stages; two genes encoding proteins involved in amino acid metabolism (PY04370 and PY05086) differentially expressed in RAS only. Similarly, PY00695, which encodes a protein that may modulate the immune response, was up regulated in 24hrLS, more than RAS, wtSPZ or 48hrLS, compared to the blood stages. Additionally, most genes encoding proteins involved in protein trafficking and DNA synthesis were differentially expressed in 24hrLS.

### Validity of Microarray results

**Figure 2:**
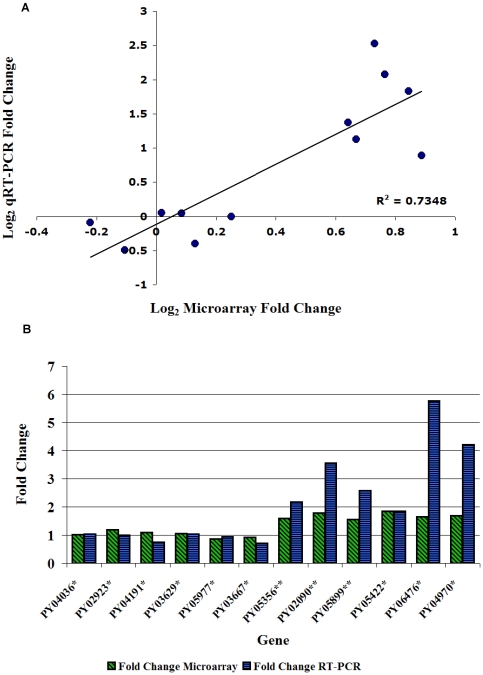
Validation of microarray results. Quantitative real time PCR (qRT-PCR) was performed on select genes found to be differentially expressed in the microarray analysis. A. The log2 of fold change in the microarray was plotted against the log2 of fold change derived from real time PCR to estimate the correlation between the microarray results and the real time PCR results. B. Actual fold change from microarray analysis (green bars) and qRT-PCR (blue bars) were graphed side by side. * fold change was calculated wtSPZ expression divided by RAS expression, ** fold change was calculated 24hrLS expression divided by 48hrLS expression.

To validate the results from the microarray, real time qRT-PCR was performed on 12 genes detected by microarray analysis. Genes were randomly selected for real time qRT-PCR from the list of genes considered significantly differentially expressed based on the SAM results. An exception was PY05422 which was selected based on its putative function. The correlation between genes assayed by real time PCR analysis and by microarray analysis was significant (R^2^ = .73, Figure 2A).Microarray analysis tended to underestimate the fold change, especially in the liver stage samples (Figure 2B).

### Pre-erythrocytic stage Transcriptome comparisons

The individual constituents of the pre-erythrocytic stage of *Plasmodium* behave differently within the mammalian host. The molecular biological events that underlie these phenotypic differences are not entirely known but are likely to be reflected in the transcriptional profile of the pre-erythrocytic stage constituents. To identify genes differentially expressed between the various constituent members of the pre-erythrocytic stages, SAM analysis was performed comparing the gene expression of each constituent of the pre-erythrocytic stages to each of the other constituents individually.

**Figure 3:**
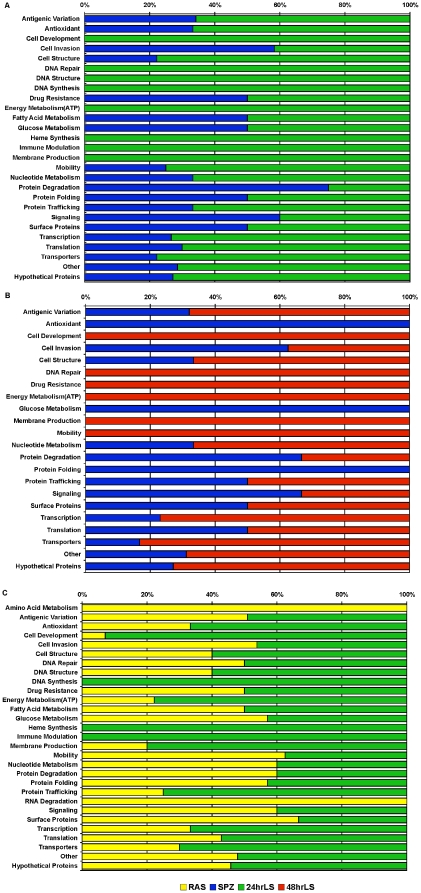
Transcriptional comparisons of pre-erythrocytic stage constituents. Statistical Analysis for Microarray (FDR <10%) was used to identify genes significantly differentially expressed when the wild type sporozoite (SPZ) transcriptome was compared with the transcriptome of 24 hr liver stage parasites (24hrLS). The significantly differentially expressed genes were placed into functional groups based on the annotated functions of the encoded proteins. The percent of the total number of genes within a particular functional group that are upregulated in wtSPZ compared to 24hrLS are graphed in blue bars and the percentage upregulated in 24hrLS relative to wtSPZ is graphed in green bars. Similar comparisons were made between B. wtSPZ, blue bars, and 48hr liver stage parasites (48hrLS), red bars, as well as between C. radiation attenuated sporozoites (RAS), yellow bars, compared with 24hrLS, green bars.

Similar to the results of transcriptional comparisons between the pre-erythrocytic stages and the mixed blood stages, most of the genes that were found to be significantly differentially expressed between any two pre-erythrocytic stage constituents encoded hypothetical proteins. Overall, the largest number of differentially expressed genes was found when wtSPZ or RAS were compared to the liver stages. For example, SAM analysis comparing wtSPZ and 24hrLS, identified 569 genes that were differentially expressed between the two with a false discovery rate (FDR) of <10%. Grouping the differentially expressed genes into functional categories (Figures 3A, blue bars and Table S2) indicated that wtSPZ up regulated genes encoding proteins functioning in cell invasion (7), protein degradation (3) and signal transduction (3). Additionally, Figure 3A, (green bars, detailed in Table S2) shows that several genes were up regulated specifically in 24hrLS parasites including some encoding proteins functioning in translation (7), transcription (11), protein trafficking (2), nucleotide metabolism (2), ATP production (6), membrane production (3), immune modulation(1), heme synthesis (3), DNA synthesis (1), repair (1) and structure (2), cell development (7), and a variety of transporters (7).

**Figure 4:**
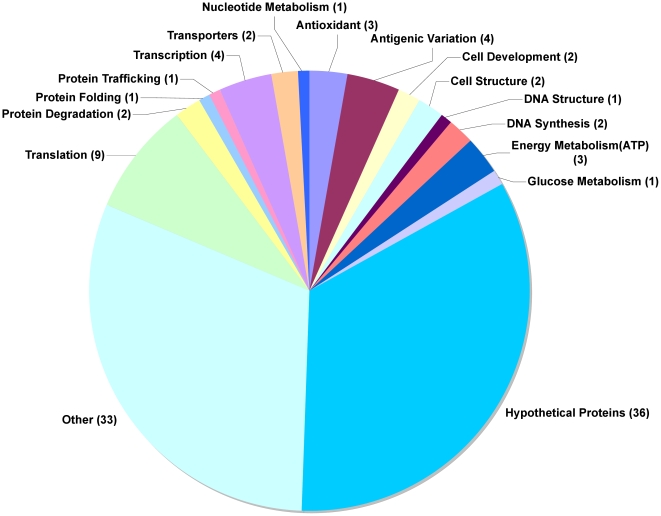
Comparison of the 24hr and 48hr Liver Stage Transcriptomes. SAM analysis (FDR <14%) identified 107 genes differentially expressed in the 24 hr liver stage transcriptome compared to the 48 hr liver stage transcriptome. All differentially expressed genes were up regulated in 24hrLS compared to 48hrLS parasites. The numbers in parenthesis represent the number of genes in a particular functional group.

SAM analysis identified 361(FDR <10%) genes as significantly differentially expressed between wtSPZ and 48hrLS parasites (Figure 3B, Table S3). Genes encoding proteins involved in cell invasion (5), glucose metabolism (2), protein folding (2), and antioxidant defense (2) were up regulated in wtSPZ relative to 48hrLS (Figure 4B, blue bars). Conversely, 48hrLS parasites (Figure 3B, red bars; Table S3) up regulated genes encoding proteins functioning in nucleotide metabolism (2), mobility (3), ATP production (3), DNA repair (1), and cell development (10). Overall, the pattern of differential gene expression between wtSPZ and 24hrLS (Figure 3A, Table S2) as well as RAS and 24hrLS (Figure 3C, Table S5) were the same. Similarly, the overall pattern of differential gene expression was similar between wtSPZ and 48hrLS (Figure 3B, Table S3) and RAS and 48hrLS (data not shown, Table S6).

The transcriptomes of RAS and wtSPZ were the most similar, with only 12 genes significantly differentially expressed between the two (FDR <10%) (Table 1). All twelve of these genes were up regulated in wtSPZ relative to RAS. Among the up regulated genes were five encoding hypothetical proteins as well as genes encoding a helicase (1), a phosphatase (1), rhoptry protein (1), putative members of the YIR protein family (2) a homeodomain containing protein (1), and superoxide dismutase (1).

**Table 1:** Annotations of genes differentially expressed in wtSPZ compared to RAS.

Gene ID	Annotation	Features	Function	Fold change
PY01902	DEAD/DEAH box helicase		Multifunctional, unwinding of DNA double strand	1.79
PY04274	Homeobox-containing protein		Regulation of patterns of development during cellular morphogenesis	1.57
PY04964	Yir2 protein		Putative variable surface antigen	2.06
PY04970	1-deoxy-D-xylulose 5-phosphate synthase		Steroid biosynthesis	1.70
PY05197	Hypothetical protein			1.44
PY05422	Superoxide dismutases		Antioxidant defense	1.85
PY05941	Hypothetical protein			1.75
PY06056	Yir4 protein		Putative variable surface antigen	1.30
PY06378	Hypothetical protein			1.58
PY06476	Hypothetical protein	signal sequence and TM domain		1.77
PY06945	Rhoptry protein		Cellular invasion	1.77
PY07504	Hypothetical protein	signal sequence and TM domain		1.44

Annotations were obtained as described in methods. Fold change ratio of average gene expression in wtSPZ relative to RAS.

TM: Transmembrane domain, Yir: yoelii interspersed repeat.

Comparing the 24hrLS and 48hrLS transcriptomes (Figure 4, Table S4), we identified 107 differentially expressed genes (FDR <14%), all of which were up regulated in 24hrLS compared to 48hrLS. Among the up regulated genes were thirty-eight genes encoding proteins with unknown functions. An additional twenty-four encoded proteins with multiple potential functions. The remaining forty-three differentially expressed genes, encoded proteins involved in several functions including antigenic variation (4), nucleic acid metabolism (1), regulation of chromatin (1), transcription (4), solute transporters (3), protein degradation (2), protein synthesis (9), and membrane fusion (3). There were also, several kinases (2) and proteases (3) up regulated in 24hrLS compared to 48hrLS.

### Hierarchical Cluster Analysis

**Figure 5:**
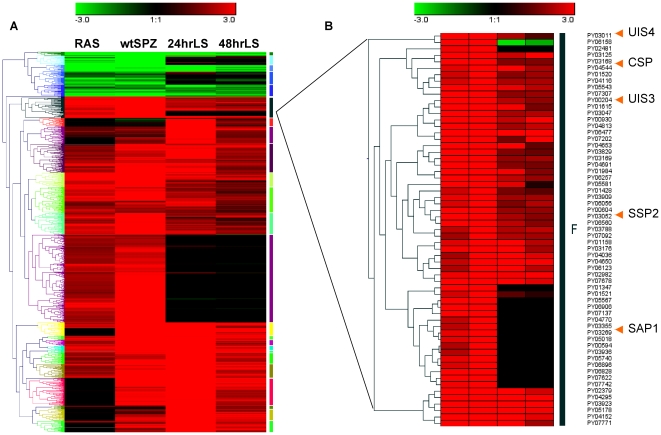
Hierarchical cluster analysis. A. Dendrogram of hierarchical clustering of the gene expression profiles of all genes found to be differentially expressed in any or all of the pre-erythrocytic stage samples tested relative to the mixed blood stages. The colored bars to the right indicate the resulting twenty-three clusters. Biological relevance of the resulting clusters was assessed by comparing the annotated functions of members of several clusters, for example Cluster F shown in panel B.

A large portion of the genes identified in this analysis encoded hypothetical proteins. Since genes with similar functions tend to have similar expression patterns [15]–[17], we performed hierarchical cluster analysis on all genes found to be differentially expressed based on their expression relative to the mixed blood stage. This resulted in the grouping of genes into 25 clusters, ranging in size from 10 to 262 genes (Figure 5A, Table S1).

For example, Cluster F (Figure 5B) contained 61 genes, several of which are known to participate in sporozoite invasion of hepatocytes and development of *Plasmodium* during the liver stage [18]–[22]. For example, PyUIS4 (PY00204), PyUIS3 (PY03011), PyCSP (PY03168), and PySSP2 (PY03052) were all found together in Cluster F (Figure 5B). Also, PySAP1 (PY03269), which has recently been shown to regulate expression of several genes [22], including PyUIS3 and PyUIS4, was also found in Cluster F.

## Discussion

At several points during the complex life cycle of *P. yoelii*, the parasite undergoes several morphological changes to biochemically and immunologically adapt to internal environment of the mosquito vectors and mammalian hosts. While the published reports on *Plasmodium* blood and mosquito stages provide details of the molecular profiles of these parasite stages within their hosts, a paucity of the information on the intra-hepatic forms of the parasite exist. Yet, the pre-erythrocytic stages of *Plasmodium* have been shown to elicit the induction of protective immunity against malaria. However, attempts at identifying genes encoding protective antigens have been hampered by difficulties in obtaining RNA and proteins from purified parasite liver stages with the minimum contamination with hepatocyte-derived material. Several recently described systems have been used to overcome this problem including the utility of LCM, collection of *in vivo* infected hepatocytes via FACS sorting [23] and cDNA subtraction to remove host transcripts [24].

The purpose of this study was to gain insights into the molecular events occurring during the pre-erythrocytic stages of *Plasmodium.* To this aim we undertook microarray based comparative transcriptional analysis on radiation attenuated sporozoites (RAS), salivary gland sporozoites (wtSPZ) and liver stage parasites collected 24 hours (24hrLS) and 48 hours (48hrLS) after salivary gland sporozoite infection. Employing a common reference design, we were able to identify ∼1100 genes significantly differentially expressed during one or more of the pre-erythrocytic stages relative to the mixed blood stages. Analysis of all genes differentially expressed in each pre-erythrocytic stage constituent relative to mixed blood stages (based on the annotated functions of their encoded proteins) allowed for the comparison of the cellular functions active during each part of the pre-erythrocytic stage with the blood stages. RAS and wtSPZ were the most different from the mixed blood stages, followed by 24hrLS, with 48hrLS being the most similar to the mixed blood stages. This pattern of gene expression suggests that as the parasite progressed through the pre-erythrocytic stage, its transcriptional profile shift from “sporozoite-like” to “blood-stage-like”.

Collectively, these results could be indicative of two possibilities. One, the biological processes required for progression through the pre-erythrocytic stages are so different from those needed for erythrocytic stage progression that different genes/proteins are required for each portion of the life cycle. Alternatively, although some of the biological processes required for development through the pre-erythrocytic and erythrocytic cycles are similar, they occur in distinct environments, which necessitate the use of distinct sets of genes/protein to be carried out efficiently. Taken together our data set supported the latter. For example several members of the *yir* gene family, encoding *Yoelii* Interspersed Repeat (YIR) proteins (Figure 1, antigenic variation), a large family of surface proteins thought to play a role in immune evasion [25], appeared to transition from a set of transcripts up regulated by RAS and wtSPZ to a distinct set of transcripts in up regulated 48hrLS. The YIR members up regulated by 24hrLS were a mixture of those expressed by RAS/wtSPZ and 48hrLS (Table S1). This could indicate that wtSPZ and RAS are subjected to antibody responses on their way to the liver. After successful intrahepatic development into merozoites, the YIR family members are changed to facilitate the parasite survival during invasion of red blood cells upon merozoite release into the blood stream.

Sixty percent of the genes found to be differentially expressed compared to the blood stages encoded proteins with no known function. Since genes encoding proteins involved in similar biological processes tend to have similar expression profiles [16], [17], [26], we performed hierarchical cluster analysis to gain insight into the possible function of the hypothetical proteins. Several of the resulting clusters contained genes encoding proteins with similar annotated functions, along with many genes encoding hypothetical proteins. Further characterization of genes encoding hypothetical proteins in the context of the dominant functional groups represented in the clusters they are grouped may shed light on their biological roles during the pre-erythrocytic stages.

Interestingly, we identified PY00695 to be up regulated in 24hrLS more than RAS, wtSPZ or 48hrLS, when compared to the blood stages. PY00695 encodes a protein with a predicted N-terminal signal sequence, central integrin alpha N-terminal domain, and C-terminal transmembrane domain. There are homologues of PY00695 in other *Plasmodium species*, including *P. falciparum*, *P. vivax*, and *P berghei*. PY00695 is also homologous to the mouse protein (NP_082283.1), T-cell immunomodulatory protein (TIP), which was shown to protect mice from T cell mediated pathology and death in a graft versus host disease model [27]. Protein sequence comparisons indicate that the Plasmodium PY00695 homologues share similar domain organization (data not shown). If PY00695 plays a similar role during liver stage development, it could contribute to the lack of T cell responses directed at infected hepatocytes following sporozoite infection.

Comparisons were also made between each constituent form of the pre-erythrocytic stages to and each other constituent. The transcriptional profiles of the liver stages were least similar to that of wtSPZ and RAS. Encouragingly, the majority of the differentially expressed genes identified in our data set involved in cell invasion were up regulated in sporozoite (both wtSPZ and RAS) when compared to the liver stages. Also, the majority of differentially expressed genes involved in cell development, cellular structure, and membrane production were up regulated in the liver stages when compared to sporozoites (both wtSPZ and RAS). Furthermore, the majority of the differentially expressed genes involved in DNA structure and DNA synthesis were up regulated in 24hrLS when compared to either wtSPZ or RAS. Collectively, these results reflect the difference between the invasive phenotype of wtSPZ and RAS when compared to the replicative and growth phenotypes of the liver stages. The biological consequences of the up regulation of the genes in the other functional categories during the pre-erythrocytic stages (i.e. solute transport and heme synthesis in the liver stages or signaling molecules and protein degradation up regulated in sporozoites) are not presently known.

The protective efficacy and induction of sterile immunity following immunization with RAS are major drives for development of vaccines the target the pre-erythrocytic stages. The maintenance of RAS hepatocyte infectivity but inability to continue the normal intrahepatic developmental program potentially provides a fascinating biological window into the gene expression repertoire and a key for vaccine development. A recent study [28] comparing *P. falciparum* transcripts of non-irradiated and irradiated (15,000 GY) sporozoite demonstrated various degrees of reduction of two DNA repair genes, PfUIS3, the *P. falciparum* ortholog of PyUIS3, Pf18S, PfCSP, and PfSSP2/TRAP. In our data set, the transcriptional profiles of RAS and wtSPZ identified 13 genes that were down regulated in RAS relative to wtSPZ, two of which encode proteins with annotated functions involved in DNA damage repair. The high degree of overlap between the gene expression profiles of RAS and wtSPZ was unexpected and could arise for several reasons. One, the gamma irradiation induced DNA damage may not lead to any transcriptional changes but rather the DNA strand breaks induced by gamma irradiation may render the parasite genome structurally incapable of replication, thus preventing intrahepatic development. Two, since both RAS and wtSPZ can migrate to the liver and invade hepatocytes, changes to the transcriptional profile of RAS may not occur until after the RAS contact and/or invade hepatocytes. Once inside hepatocytes, wtSPZ can develop into merozoites but RAS cannot. In fact, it has been recently shown that the transcriptional profile of P. *falciparum* sporozoites is altered upon hepatocyte contact [29]. Finally, the small differences in the transcriptional profiles of wtSPZ and RAS may translate into biologically significant changes. The genes encoding proteins involved in DNA repair down regulated in RAS relative to wtSPZ could indicate that RAS is not able to repair the damage to its genome caused by irradiation. Further characterization of the liver stage parasites derived from RAS would shed light on the mechanism of parasite intrahepatic developmental arrest. Due to their very minute size arising from their developmental arrest we failed in our attempts to obtain sufficient RNA for microarray analysis from 24hr and 48hr RAS-derived liver stage parasite. We hope that newer systems that allow microarray analysis with nanogram quantities of RNA provide a means for transcriptional profiling of the RAS-derived liver stages.

In general, the transcriptional profiles of the 24hr and 48hr liver stage parasites were similar as well. The fact that the observed phenotypes of 24hrLS and 48hrLS parasites are different, yet transcriptionally they are similar suggests that the majority of the genes required for liver stage development get turned on early during hepatocyte invasion. This implies that a post-transcriptional method of regulation may control the transition from the DNA synthesis and growth phenotype of 24hr liver stage parasites to the cytokinesis and merozoite formation phenotype of 48hr liver stage parasites. All of the differentially expressed genes identified by comparing the two liver stage time points were up regulated in 24hrLS parasites compared to 48hrLS parasites. Further characterization of the up regulated genes could reveal the role they play in the biological processes needed to facilitate the very high levels of DNA synthesis and growth in size observed 24hr after sporozoite invasion of hepatocytes.

There have been previous attempts to perform global gene expression analysis on the pre-erythrocytic stages of *P. yoelii* [23], [24], [30]. Previously, our lab created a cDNA library from LCM collected late liver stage schizonts. Comparison between the data set presented here and the previously published cDNA library revealed that only 53 out of 623 total genes from the LCM library were identified as significantly expressed in the liver stage microarray analysis presented here. This result is likely due to the fact that cDNA libraries are not quantitative and/or the fact that a limited number of random clones from the cDNA library were sequenced. Thus some of the transcripts identified in the LCM library may represent low abundance transcripts that would have been excluded from microarray analysis.

There are similarities and differences among the various pre-erythrocytic stage transcriptional data sets. For example, both the data set presented here and that of Tarun, et. al. found that the transcriptomes of the 24 hour and 48 hour liver stage parasites were more similar to each other than to that of either the mixed blood stages or to salivary gland sporozoites. Also, each of the three liver stage transcriptomes contained genes encoding proteins involved in type II fatty acid synthesis which has been shown to be important in the development of *Plasmodium* within hepatocytes [31]–[35]. However, different total numbers (Tarun, et. al.: 17 and Zhou, et. al.: 18 and Williams, et. al.: 4) of known members of the gene family were identified in each data set. The differences between the various pre-erythrocytic transcriptional data sets result from a combination of different sample collection and processing protocols, as well as differences in raw data acquisition and processing methodology used to create the various gene lists.

Comparing the pre-erythrocytic stage microarray data sets overall (Table S8), there were 794 genes expressed above threshold in wtSPZ in the dataset presented here compared to 2246 genes detected in the expression array data set of Zhou et. al. [24] with an overlap of 329 genes between the two data sets (p<.376). A total of 617 genes were identified as expressed above threshold in the liver stages in the data set presented here with an overlap of 234 genes (p<.240) to the 2161 genes identified as expressed in the liver stage within the expression array data set of Zhou et. al. [24] Also, there was an overlap of 238 (p<2.045e-15) between the 1951 genes identified as active during the liver stage in the data set of Tarun et. al. [23] and the 515 genes differentially in the liver stages relative to the mixed blood stages in the data set presented here.

At present, it is hard to interpret the significance of the overlap between gene lists from different microarray experiments [36]–[39]. Therefore, each of the individual data sets may offer a snapshot of the gene expression during the pre-erythrocytic stages under a particular set of experimental circumstances. Presently, consensus has not been reached regarding the appropriate method for combining the results of multiple microarray studies, while taking into account the differences in experimental methodology, to obtain improved understanding of the overall pattern of differentially expressed genes. Thus, researchers interested in studying specific genes during the pre-erythrocytic stages would be well served to compare the expression in each of the data sets and perform confirmatory studies, such as PCR or northern blots, to guide their studies.

Contained within the data set presented here are many genes encoding proteins that participate in a variety of biological processes that are important for parasite progression through the pre-erythrocytic stages and development into merozoites. Further characterization of the many genes expressed during the pre-erythrocytic stages could lead to further insights in to the biology of the pre-erythrocytic stages and ultimately, provide new targets for use in anti *Plasmodium* drug and vaccine development.

## Methods

### Sporozoite collection

*P. yoelii* infected *Anopheles stephensi* mosquitoes were provided by Dr. Fidel Zavala (Johns Hopkins Bloomberg School of Public Health), 18–21 days post infection. Some *P. yoelii* infected mosquitoes were subjected to 12,000 rad of gamma irradiation. Both irradiated and non-irradiated infected mosquitoes were anesthetized by incubation at −20°C and processed separately as follows. Mosquitoes were washed in 70% ethanol and twice in M199 media (GIBCO) containing 5% heat inactivated FBS (Invitrogen). The thorax and heads of washed mosquitoes were separated from the abdomen, collected and ground into slurry using a mortar and pestle. Sporozoites were collected from slurry using a renografin gradient (Bracco Diagnostics), and suspended in either M199 media containing 3% naïve mouse serum for injection into BALB/c mice or in lysis solution (RNAqueous Micro kit, Ambion) for RNA extraction.

### Mouse infections, liver harvest and sectioning

Two groups of four naive 4–6 week old female BALB/c mice (Charles River Laboratories) were intravenously injected with approximately 1×10^6^ sporozoites. Infected mice were euthanized by overdose with 2.5% Avertin (Sigma) and had their livers removed either 24 hrs or 48 hrs after sporozoite infection as previously described^13^. Livers were washed in sterile 1XPBS and placed into a cryomold containing OTC freezing medium (Sakura). To freeze livers, cryomolds containing livers were partially immersed in a beaker of 1-butonal which was itself partially submerged in liquid nitrogen. Frozen livers were placed at−80°C until used. A cryostat (Leica) was used to cut 7 µm thick frozen tissue sections from the liver portions stored in OTC, all sections are stored at −80°C until used. The dissection and freezing process took less than ten minutes to complete. All mice were housed in a pathogen-free facility and were tested routinely for mouse hepatitis virus and other pathogens. All mice were handled according to protocols approved by the University of Maryland School of Medicine Institutional Animal Care and Use Committee. The procurement, conditioning/quarantine, housing, management, veterinary care, and disposal of carcasses followed the guidelines in the NIH Guide for Care and Use of Laboratory Animals.

### Staining of liver sections

Liver sections were fixed in ice cold 75% ethanol and stained using FITC-tagged monoclonal antibody NYLS3, which has been previously shown to bind to the *P. yoelii* antigen, HEP17 [40], diluted in LCM staining solution. LCM staining solution consisting of RNAse inhibitors (Superase, Ambion) added to a 1∶50 mixture of staining solution (Arcturus) in 1XPBS was used to prevent degradation of RNA during staining of sections. Sections were washed twice with LCM staining solution to remove excess antibody and then were immediately dehydrated according to Histogene LCM Immunofluorescence Staining Kit protocol (Arcturus). Following dehydration sections underwent LCM as previously described [30].

### Laser capture microdissection

Briefly, parasites were visualized using inverted fluorescent microscope equipped with a low power infrared laser (PIXCell IIe, Arcturus). A transparent, thermosensitive cap was placed on top of the tissue section and the laser was fired, embedding the tissue containing the parasite into the membrane. The cap was removed from on top of the tissue section, leaving the majority of the host tissue attached to the microscope slide. The tissue containing the parasite remains attached to the membrane. This procedure was repeated several times per section, allowing roughly 5–25 parasites to be collected per section. Only infected hepatocytes collected within 90 minutes of dehydration were used for RNA extraction.

### RNA extraction and linear amplification

#### Whole Livers

Portions of livers harvested from mice infected with *Plasmodium yoelii* were homogenized using glass tissue homogenizer. Total RNA was collected from liver homogenate using Trizol reagent (Invitrogen). 10 µg total RNA was DNAse treated using DNAfree (Ambion). DNAse free total RNA was subjected to one round of linear amplification (Ambion) and the resulting aRNA was converted in to cDNA using AffinityScript QPCR cDNA Synthesis Kit (Stratagene). This cDNA was used qRT-PCR reactions, as all of the LCM collected material was used in the microarray hybridizations.

#### LCM material and sporozoites

The membranes for LCM caps containing captured infected hepatocytes were removed and submerged in lysis solution from RNaqueous micro kit (Ambion) at room temperature for five minutes. The membranes were removed and total RNA was extracted from the resulting lysate following manufacturers' instructions. Total RNA was then DNAse treated using DNAfree kit following manufacturers' instructions. Total RNA was extracted from two groups of 2×10^7^ gradient purified wild type sporozoites or radiation attenuated sporozoites using RNaqueous micro kit (Ambion).

#### Linear amplification

Total RNA was subjected to one round of T7 based linear amplification using the amino Allyl Message Amp II amplification kit (Ambion) according to the manufacturer's protocol. The resultant amplified RNA (aRNA) was labeled, to yield fluorescent Cy3 and Cy5 labeled aRNA. Also, aRNA generated from total RNA extracted from *P. yoelii* mixed blood stage parasites was labeled simultaneously and used as a reference sample for all hybridizations.

### *P. yoelii* genomic microarray hybridization

*Plasmodium yoelii* oligonucleotide microarrays were produced in the Molecular Genomics Core Facility, Drexel University College of Medicine. These microarrays are spotted with 65 base pair oligonucleotides, with each spot representing one of the ∼6700 open reading frames predicted from the analysis of the *P. yoelii* genomic sequence [41]. The arrays are arranged into 32 quadrants with each quadrant containing duplicate *P. yoelii* spots (containing oligonucleotides from a single *P. yoelii* gene).

*P. yoelii* blood stage RNA was used as a reference standard because multiple samples were going to be collected at different times and liver stage RNA collected by LCM could not be collected in sufficient quantity to allow multiple direct comparisons on the array. Therefore, all samples were compared to a reference RNA sample. Labeled aRNA was purified and fragmented using fragmentation reagents from Ambion. The labeled fragmented aRNA was mixed polyA RNA (Invitrogen), 0.2% SDS, 20 mM Hepes, pH 7.2, in 3′ SSC and hybridized in a 1∶1 ratio of experimental to reference aRNA to a microarray at 65°C for 16 hours. After hybridization, the microarrays were washed in SSC buffer with increasing stringencies and dried. Next each microarray was scanned using a GenePix 4000A laser scanner and the array features (spots) were quantified using the GenePixPro 5.0 and normalized using the Acuity 3.0 Microarray Informatics Software (Axon Instruments). Each microarray contained several control spots positive control spots (containing olignucleotides from several *P. yoelii* genes), mouc+ spots (containing oligonucleotides from mouse genes), several different negative control spots (containing oligonuclotide with random sequences), empty spots (containing no oligonucleotides) and 3xSSC spots (containing 3xSSC). The intensities of these control spots were used to normalize the whole array and to calculate a threshold value. Results of all the spots in a quadrant were excluded if the average intensity of the positive control spots was lower than the average intensity of the empty spots and/or the 3xSSC spots. Genes were considered expressed if their average intensity across all arrays was greater than or equal to 1.5 times the threshold value. Enough aRNA was not generated for dye swaps to be performed for all the liver stage samples, they were all labeled with Cy3 with the exception of one dye flip hybridization performed for a single biological 48hrLS sample. Six hybridizations including two dye flip hybridizations were performed with both wild type and radiation attenuated sporozoites. Seven total hybridizations representing four independent biological replicates from 24 hr liver stage parasites and six total hybridizations representing four independent biological replicates from 48 hr liver stage parasites were performed. Results of microarray hybridizations were deposited in the Gene Expression Omnibus, accession number: GSE18730. All data is MIAME compliant as detailed on the MGED Society website: http://www.mged.org/Workgroups/MIAME/miame.html.

### Assessment of differential gene expression

Significance Analysis of Microarray [14] (SAM) was used to identify the genes which were differentially expressed, SAM analysis determines the statistical significance of changes in gene expression by calculating an observed score based on the average change in gene expression relative to the standard deviation of multiple measurements of expression for that gene. Using a permutation-based method; SAM calculates an expected score for each gene, representing the random fluctuation in gene expression when there is no real significant difference in gene expression. When the difference between the observed and expected scores is beyond a certain threshold (delta), the change in gene expression is called significant. For each delta value, SAM estimates the false discovery rate (FDR) from the average number of falsely significant genes in all the permutations divided by the number of permutations. Finally, for each gene SAM computes a q-value that quantitatively measures how significantly the gene is differentially expressed and is similar to the p value but is adapted to the analysis of large numbers of genes common in microarray studies [12], [13].

For this study SAM was first used to measure the significance of differential gene expression for each gene by comparing expression in the sporozoite, RAS, 24 hour liver stage or 48 hr liver stage to expression in the blood stage. Two thousand permutations were used and delta was set for each comparison to keep the FDR at <1%. Next, SAM was used to compare the gene expression of each member of the pre-erythrocytic stage of *Plasmodium* to each other member. In these comparisons, two thousand permutations were used with FDR set to <10% for all comparisons except when comparing the 24hrLS to the 48hrLS for which FDR was set at <14%. For these comparisons, the ratio of gene expression in the pre-erythrocytic stage of each gene to its expression in the blood stage was used, as the arrays employed in this study were not designed for direct comparison of one array to another. For example, 24 hour liver stage gene expression relative to expression in the blood stage was compared to 48 hour liver stage gene expression relative to the blood stage, then to sporozoites relative to the blood stage and finally to RAS relative to the blood stage.

### Cluster Analysis

The observed score (see above) from all genes found to be differentially expressed compared with the blood stage by SAM analysis were used to perform hierarchical cluster analysis using the Genesis program [42]. The observed score was used instead of ratios because the observed score takes into account fluctuations in the data resulting from multiple measurements, providing a better measurement of gene expression across all hybridizations. Hierarchical clustering was performed using complete linkage and euclidean distance. The validity of cluster predictions was probed by determining if some the genes in each cluster encode protein with similar annotated functions (PlasmoDB.org).

### Functional groupings

Genes found to be significantly differentially expressed in wild type sporozoites compared to mixed blood stages were placed into functional categories based on their annotated functions. *P. yoelii* annotations were obtained from PlasmoDB and/or the result of BLAST searching using the protein sequences encoded by the differentially expressed genes compared to all available *Plasmodium* genomes on PlasmoDB.org and NCBI. Genes differentially expressed in RAS and the liver stages were similarly grouped.

### Real time RT-PCR

Several genes identified in the microarray were subjected to real time PCR to validate the results of the microarray. Genes were randomly selected for real time RT-PCR from the list of genes considered significantly differentially expressed relative to the mixed blood stages based on the SAM results but prior to functional group assignment and cluster analysis. An exception was PY05422 which was selected based on its putative function. Primers were designed using MacVector (MacVector, Inc.) and synthesized at the Biopolymer Core facility (UMB), primer sequences are listed in Table S7. Primers were designed to be 18–30 bps in length, GC content 30–60%, amplify region 50–200 bp long and when possible, to bind within 600 bp of the 3′ end of the genes of interest. Primer efficiencies were all between 89–105%, and qRT-PCR resulted in no detectable primer dimmers. The Brilliant® II SYBR® Green QRT-PCR AffinityScript™ Master Mix, 2-Step kit (Stratagene), was used to produce cDNA from 24hrLS, 48hrLS, RAS and wtSPZ total RNA derived aRNA, cDNA was diluted 1∶5 with water and 2 ul diluted cDNA was used in 12 ul reactions qPCR reactions performed on a Mx3000 qPCR system (Stratagene). qPCR conditions were as follows, 94°C for minutes followed by 40 cycles of 94°C secs, 55°C 15 secs, 72°C 30 secs, followed by dissociation curve. Two or three biological replicates, with three technical replicates from each biological replicate were performed for each assayed gene. Fold change was determined by the Plaffl method [43] using PyHsp70 (PY06158) as a reference gene for RAS, wtSPZ, 24hrLS, and 48hrLS. Primer sequences are found in Table S7.

### Microarray Data Set Comparison

The web based calculator, statistical significance of the overlap between two groups of genes, was used to compare the gene lists presented in this manuscript with either the gene list generated by Zhou et. al. [24] or Tarun, et. al. [23] This calculator uses the total number of genes common to each of the microarray platforms in the one-sided hypergeometric test to determine if the number of genes common between the data set presented here and either the Zhou et. al. [24] or the Tarun, et. al. [23] data sets arose by chance. *P. yoelii* genes that did not have probes on both microarrays being compared were excluded. Also, genes found in any data set that has a Gene ID that does not match with a *P. yoelii* gene ID from Plasmodb.org was excluded as well. The list of all *P. yoelii* genes identified in any of the microarray data sets discussed above can be found in supplemental Table S8. The calculator was developed by Jim Lund at the University of Kentucky and is found at: http://elegans.uky.edu/MA/progs/overlap_stats.html.

## Supporting Information

Figure S1 Distribution of P. yoelii Genes Differentially Expressed throughout the Pre-erythrocytic Stage Relative to the Mixed Blood Stages. P. yoelii genes that were found to be differentially expressed throughout the pre-erythrocytic stage relative to the mixed blood stages were grouped based on the annotated function of their encoded proteins.
(0.55 MB TIF)

Table S1 List of Genes Differeitally Expressed During the Pre-Erythrocytic Stage Relative to the Mixed Blood Stages.
(0.35 MB XLS)

Table S2 List of Genes Differentially Expressed in 24hrLS relative to wtSPZ.
(0.21 MB XLS)

Table S3 List of Genes Differentially Expressed in 48hrLS relative to wtSPZ.
(0.23 MB XLS)

Table S4 List of Genes Differentially Expressed in 24hrLS relative to 48hrLS.
(0.15 MB XLS)

Table S5 List of Genes Differentially Expressed in 24hrLS relative to RAS.
(0.24 MB XLS)

Table S6 List of Genes Differentially Expressed in 48hrLS relative to RAS.
(0.21 MB XLS)

Table S7 List of Primers used in RT-PCR analysis.
(0.03 MB DOC)

Table S8 Comparison of Pre-erythrocytic Stage Microarray Results.
(0.69 MB XLS)
