# Supplementary figures and images for: Transcriptional Analysis of the Pre-Erythrocytic Stages of the Rodent Malaria Parasite, Plasmodium yoelii
Source: PLoS One. 2010 Apr 21;5(4):e10267. doi: 10.1371/journal.pone.0010267 (PMC2858153; doi:10.1371/journal.pone.0010267)

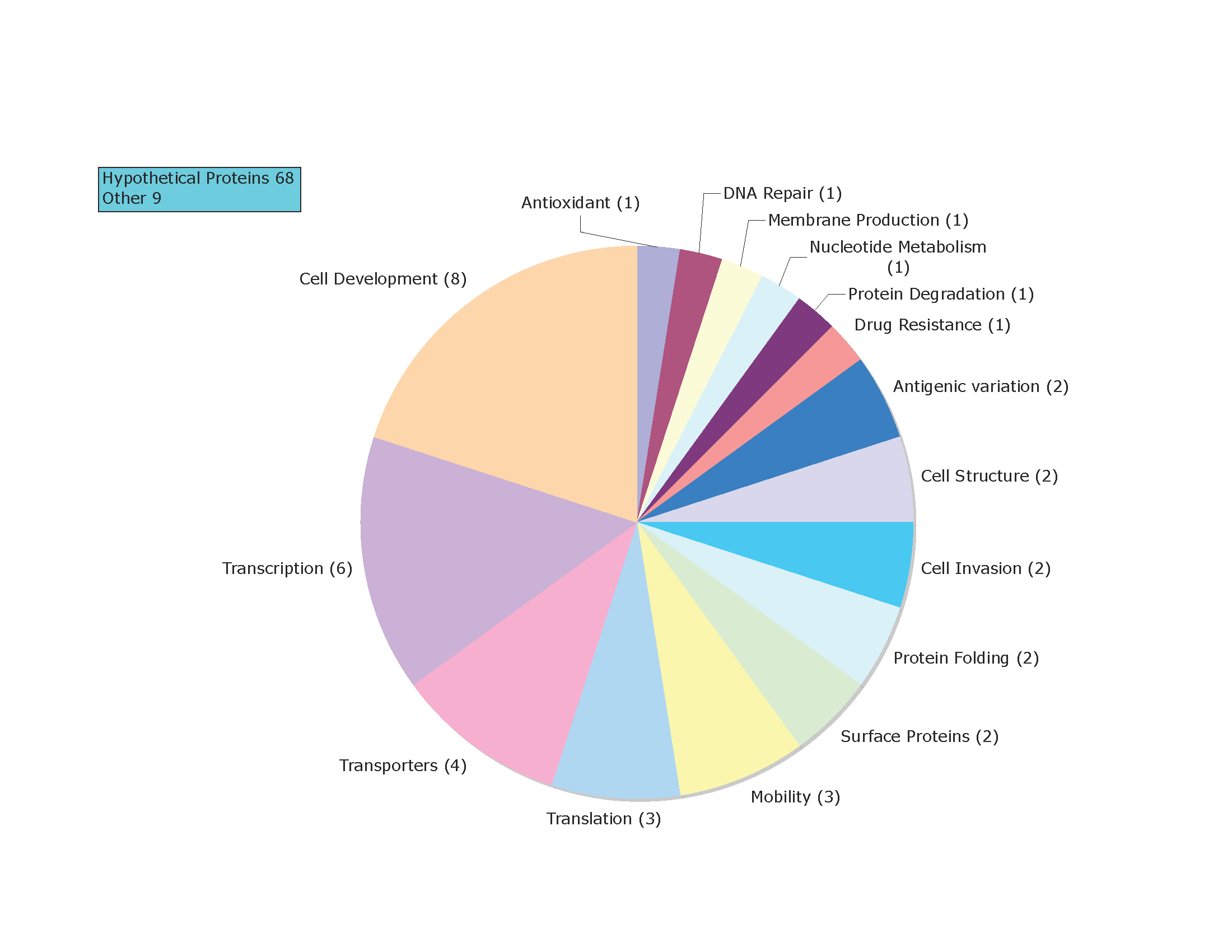

Supplement: Figure S1 — Distribution of P. yoelii Genes Differentially Expressed throughout the Pre-erythrocytic Stage Relative to the Mixed Blood Stages. P. yoelii genes that were found to be differentially expressed throughout the pre-erythrocytic stage relative to the mixed blood stages were grouped based on the annotated function of their encoded proteins. (0.55 MB TIF) [file pone.0010267.s001.tif]
