# Supplementary material for: Transcriptional Analysis of the Pre-Erythrocytic Stages of the Rodent Malaria Parasite, Plasmodium yoelii
Source: PLoS One. 2010 Apr 21;5(4):e10267. doi: 10.1371/journal.pone.0010267 (PMC2858153; doi:10.1371/journal.pone.0010267)
Supplement: Table S7 — List of Primers used in RT-PCR analysis. (0.03 MB DOC) [file pone.0010267.s008.doc]

PY02090for AAGGTGGCTCCTACTGG

PY02090rev TCTTCTCCAAAGGCG

PY02923for GGTAGTTGGGTTGACGAATTTAAC

PY02923rev CCATCCATCTGATAAACCATGAC

PY03629for TGTAGTTGTAGGAGGTGGATGTG

PY03629rev GCTGCTCTCTTGAACCCAATG

PY03667for GCTCAGGAAATGAATAATGTGTCAG

PY03667rev CGACTGTTGAAGGTCTCTGTC

PY04036for GGTTCCTGGAGATTTTCTTGC

PY04036rev AACTATTGGATGTTGGCTGTTC

PY04191for CATTTGCTTGGACCAGGAG

PY04191rev TGCTTCATCCGATTCTTCG

Py04970for AGAATTGGATAGTTGCCATGAC

Py04970rev AGAACCTCCTAACATAGCTGGAG

PY05356for CCTGTCGCAGAATCTTTTGG

PY05356rev TTGTGGAAATGCTTGACCAG

PY05422for TTCAGGATGGGGATGGTTAG

PY05422rev TGGATTTCCAGCATCATGTG

PY05899for TGGATGGGCATTGTTTGC

PY05899rev CAACAGCAACTCCAGAGACAAG

PY05977for GTGCTCCTGAACATTTTCGTAG

PY05977rev CGTTTCCTTATCACCTCATCC

PY06476for GGTTTTAAGTTCCCATCATTCC

PY06476rev GAGCCTGAGAAATAGCCAATTC

PY06158for GCTGATAACCAACCAGGAGTC

PY06158rev CAGGTGGGATACCATCTAAATG

Table S3. Primer sequences used for qRT-PCR.
